# Statistical modeling of surveillance data to identify correlates of urban malaria risk: A population-based study in the Amazon Basin

**DOI:** 10.1371/journal.pone.0220980

**Published:** 2019-08-09

**Authors:** Rodrigo M. Corder, Gilberto A. Paula, Anaclara Pincelli, Marcelo U. Ferreira

**Affiliations:** 1 Department of Parasitology, Institute of Biomedical Sciences, University of São Paulo, São Paulo, Brazil; 2 Department of Statistics, Institute of Mathematics and Statistics, University of São Paulo, São Paulo, Brazil; Instituto Rene Rachou, BRAZIL

## Abstract

Despite the recent malaria burden reduction in the Americas, focal transmission persists across the Amazon Basin. Timely analysis of surveillance data is crucial to characterize high-risk individuals and households for better targeting of regional elimination efforts. Here we analyzed 5,480 records of laboratory-confirmed clinical malaria episodes combined with demographic and socioeconomic information to identify risk factors for elevated malaria incidence in Mâncio Lima, the main urban transmission hotspot of Brazil. Overdispersed malaria count data clustered into households were fitted with random-effects zero-inflated negative binomial regression models. Random-effect predictors were used to characterize the spatial heterogeneity in malaria risk at the household level. Adult males were identified as the population stratum at greatest risk, likely due to increased occupational exposure away of the town. However, poor housing and residence in the less urbanized periphery of the town were also found to be key predictors of malaria risk, consistent with a substantial local transmission. Two thirds of the 8,878 urban residents remained uninfected after 23,975 person-years of follow-up. Importantly, we estimated that nearly 14% of them, mostly children and older adults living in the central urban hub, were free of malaria risk, being either unexposed, naturally unsusceptible, or immune to infection. We conclude that statistical modeling of routinely collected, but often neglected, malaria surveillance data can be explored to characterize drivers of transmission heterogeneity at the community level and provide evidence for the rational deployment of control interventions.

## Introduction

Malaria continues to be a major cause of morbidity and mortality in sub-Saharan Africa, South and Southeast Asia, Oceania, and Latin America, with 219 million cases and 435,000 deaths worldwide in 2017 [1]. The disease typically affects the rural poor, since urbanization tends to reduce malaria risk through improved housing, greater access to health services, and environmental changes that may limit vector abundance [2]. Indeed, malaria rates are typically lower in cities, compared to their rural surroundings, in most [3,4], although not all [5], endemic settings. Despite this, the rapidly growing urban population in developing countries bears an increasingly larger proportion of the global malaria burden because of both local transmission and importation from rural sites [6,7].

Sprawling towns and cities are heterogeneous, and so is urban malaria risk. For example, in the early 1980s the number of infective bites per person was estimated to range between <1 every three years and >100 per year across the city of Brazzaville [8]. Risk heterogeneity translates into overdispersed frequency distributions of malaria episodes per person, with few subjects experiencing a disproportionately large disease burden due to frequent reinfection [9, 10]. Low socioeconomic status, poor housing quality, and proximity to larval habitats are among the household-level factors that fuel malaria transmission in urban Africa [11,12]. Travel to rural communities is another major risk factor that entails a different set of preventive measures [13].

Surprisingly, the epidemiology of urban malaria has been little investigated in Latin America, the most urbanized region of the developing world [7,14]. Imported cases from surrounding rural sites appear to be the main contributors to malaria infections diagnosed in the city of Quibdó, Colombia [15,16], but urban malaria transmission has been documented in coastal Peru [17] and in the outskirts of major cities in Amazonian Brazil [18,19]. *Anopheles darlingi*, the main malaria vector in the Amazon Basin, is typically found in forested areas [20], but urban environments are not necessarily unsuitable for this species. Indeed, the accelerated urbanization process in the Amazon over the past few decades originated a multitude of small cities and towns characterized by poor housing and little public infrastructure and interspersed with rural spaces. Unsurprisingly, immature stages of malaria vectors can develop in many types of natural and man-made water habitats in precarious urban and peri-urban settlements across the region, including the fish farming ponds recently opened for commercial aquaculture [21–23].

Statistical modeling of routinely collected malaria surveillance data can be particularly challenging. Poisson regression models are commonly used to analyze count-type data in epidemiology, but cannot adequately fit overdispersed malaria case distributions that are typically found in endemic settings [9,10]. A variety of alternative models have been used instead, e.g. the negative binomial (NB) [9]. However, as malaria rates decline, more subjects will remain uninfected over extended periods of time, increasing the proportion of zero counts in cohort studies. Zero-inflated statistical models, such as the zero-inflated negative binomial (ZINB), usually provide a better fitting to malaria count data [10,24] and household-level malaria vector densities [25] with an excess of zero counts. The ZINB model combines the NB distribution and the logit distribution. As a consequence, it can additionally estimate the fraction of unexposed or protected individuals in the population by allowing for a mixture of two latent classes: (i) at-risk individuals who contribute cases according to the NB distribution function and (ii) not-at-risk individuals with zero cases, described by the logit component, hence termed “structural zeros”. The not-at-risk fraction of the population described by the structural zero component of the model is intrinsically free of any malaria risk and will remain uninfected irrespective of any protective measure. Importantly, this subpopulation can bias estimates of the effect of interventions for controlling and eliminating malaria [10]. A further challenge for statistical modeling of malaria surveillance data is the clustering of observations into households, where key risk factors for infection such as poor housing quality and proximity to mosquito breeding sites are shared [11,12]. Random-effects (RE-) ZINB models can account for the dependency between observations [26] but, surprisingly, have not yet been used to analyze data from community-wide malaria surveys.

Despite the dramatic decrease in the burden of malaria in Brazil in recent decades, focal transmission persists across the Amazon Basin [27]. Transmission rates are greatest in Juruá Valley, next to the Brazil-Peru border. With 0.5% of the Amazon's population, the region accounts for 18.5% of the country's malaria burden, estimated at 157,000 cases in 2016 [1]. A large proportion of infections in Juruá Valley are reportedly acquired in urban settings–up to 45% in the municipality of Mâncio Lima, compared with the country's average of 17% in 2013 [28]. Here, we characterize high-risk individuals and households by applying RE-ZINB regression analysis to overdispersed and household-clustered surveillance data. Our findings may allow for better targeting of interventions in the main malaria hotspot of Brazil.

## Material and methods

### Ethics statement

The study protocol was approved by the Institutional Review Board of the Institute of Biomedical Sciences, University of São Paulo, Brazil (CEPH-ICB 1368/17); written informed consent and assent were obtained.

### Study area and population

The municipality of Mâncio Lima covers a surface area of 4,672 km² in northwestern Brazil (S1 Fig) and comprises a single town next to the Japiim river, where nearly half of its 17,545 inhabitants reside. Streams, wetlands rich in moriche palm trees, and fish farming ponds are widespread in the town. With a typical equatorial humid climate, Mâncio Lima receives most rainfall between November and April, but malaria transmission occurs year-round. The annual parasite incidence, estimated at 436.4 cases per 1,000 inhabitants in 2016, is the highest for a municipality in Brazil [29]. Local distribution of long-lasting insecticidal bed nets (LLINs) and indoor residual spraying (IRS) with pyrethroids or propoxur are currently limited to high-risk households. The primary local malaria vector is *An*. *darlingi*, but *An*. *albitarsis s*.*l*. is also abundant across the town, especially in fish farming ponds [22,30].

The study population comprised all permanent residents in the town of Mâncio Lima enumerated by a census survey between November 2015 and April 2016. During the survey, dwellings were geo-localized and a questionnaire was applied to collect demographic, health, behavioral, and socioeconomic data. Principal component analysis was used to compute an assets-based wealth index for each household [31].

### Malaria surveillance and treatment

The study outcome was laboratory-confirmed malaria, defined as any episode of parasitemia, irrespective of parasite density or symptoms, diagnosed through active or passive case detection from 1 January 2014 through 30 September 2016. We retrieved malaria case records from the electronic malaria notification system of the Ministry of Health of Brazil (http://200.214.130.44/sivep_malaria/). Because malaria is a notifiable disease in Brazil and diagnostic testing and treatment are not available outside the network of government-run health care facilities, the database comprises the vast majority of malaria episodes confirmed by thick-smear microscopy in Mâncio Lima residents over the study period (33 months). According to a recent estimate, the electronic malaria notification system comprises 99.6% of all clinical malaria cases diagnosed countrywide [32]. At least 100 fields are routinely examined for malaria parasites under 1000× magnification by experienced local microscopists before a slide is declared negative. Partially supervised chloroquine-primaquine and artemether-lumefantrine regimes were administered to treat *Plasmodium vivax* and *P*. *falciparum* malaria, respectively [33]. A minimal interval of 28 days between two consecutive episodes was required to count the second episode as a new malaria infection; when different species were diagnosed <28 days apart, a single mixed-species infection was counted.

### Statistical methods

The R package *gamlss* [34] was used for statistical analysis (R Foundation for Statistical Computing, Vienna, Austria). The generalized additive models for location, scale and shape (GAMLSS) approach [35] was used to fit ZINB [10,24] distribution functions to malaria counts and to choose the best-fitting model. We note that the term “additive” refers to the option, provided by the GAMLSS approach but not applied here, to include nonparametric components into the linear predictors of the models. We used randomized normal quantile-quantile (Q-Q) plots and detrended normal Q-Q plots, known as worm plots, as diagnostic tools to analyze residuals [36].

Individual- and household-level explanatory variables were added to the count component of the first standard ZINB regression model. The individual-level variables entered in the multivariable models were: age (stratified as 0 [birth]-5, 6–15, 16–40, 41–60, and >60 years); sex (female vs. male); reported bed net use, either insecticide-impregnated or not, the previous night (no vs. yes); sleeping time (before 10 pm, between 10 and 11 pm, after 11 pm); and wake-up time (before 7 am, between 7 and 8 am, after 8 am). Household-level variables were: household size (<5 vs. ≥5 people); wealth index (stratified into terciles); LLIN available in the household (no, yes, unknown); IRS within the past three years (no, yes, unknown); and housing quality indicators such as incomplete walls and ceiling (no vs. yes), presence of screens in doors and windows (no vs. yes), and type of lavatory (indoors vs. outhouse). We used the R package *GoodmanKruskal* to identify significant pairwise associations between model covariates; none was found (S2 File). The multivariable model was adjusted for the covariate “follow-up duration”, the number of person-years at risk contributed by each study participant. This was calculated for the period between the date of birth or 1 January 2014, whichever was the most recent, and 30 September 2016, when the follow-up ended.

Next, to account for clustering of observations into households, household-level RE terms were also considered into the multivariable ZINB regression. Worm plot diagnostic of the RE-ZINB model indicated too large fitted variance, with many data points lying outside the 95% confidence intervals (CI) of the expected deviation. To reach satisfactory model diagnostics, we shrunk the random-effects distribution toward the overall mean [37] by decreasing the degrees of freedom originally estimated by the model to 150; further details are provided in S1 File.

We next used the random-effect predictors to characterize the spatial heterogeneity in malaria risk while controlling for potential confounders [26]. The high (low) magnitude of household random-effects predictors was used to select households with higher (lower) than average malaria incidence density. We examined the spatial distribution of households with the top 5% and bottom 5% random-effects predictors of the RE-ZINB models (here termed “hot houses” and “cold houses”, respectively) by mapping their GPS coordinates.

Given the results of the spatial analysis described above, we tested whether model fitting could be further improved by including a variable describing subjects' zone of residence, whether in the center (“urban hub”) or in the less-urbanized periphery of the town, close to the most vegetated areas. To this end, geo-localized houses were classified as centrally or peripherally situated using the computational approach described in S3 File. We next used the Akaike information criterion (AIC) to compare the quality of RE-ZINB models with and without the covariate “zone of residence”.

To further characterize study participants at no risk of malaria [10], we built additional RE-ZINB models with the following variables added to the structural zero component: zone of residence, age, sex, and follow-up duration. The following variables were initially entered in the count component: age, sex, bed net use, follow-up duration, zone of residence, household size, LLIN availability, recent IRS, presence of complete walls, and type of lavatory.

The best RE-ZINB models were selected using the strategy *stepGAICALL*.*A()* proposed by Stasinopoulos and colleagues [34] with the following steps: (a) an initial NB model was built for the count component (forward approach); (b) given this model, a model was built for the logit component (forward approach); (c) given the NB and logit models, we checked whether the terms for the logit model were needed using backward elimination; (d) given the NB and logit models, we checked whether the terms for the NB model were needed (backward elimination). The generalized AIC (GAIC) was used for model comparison.

## Results

**Fig 1:**
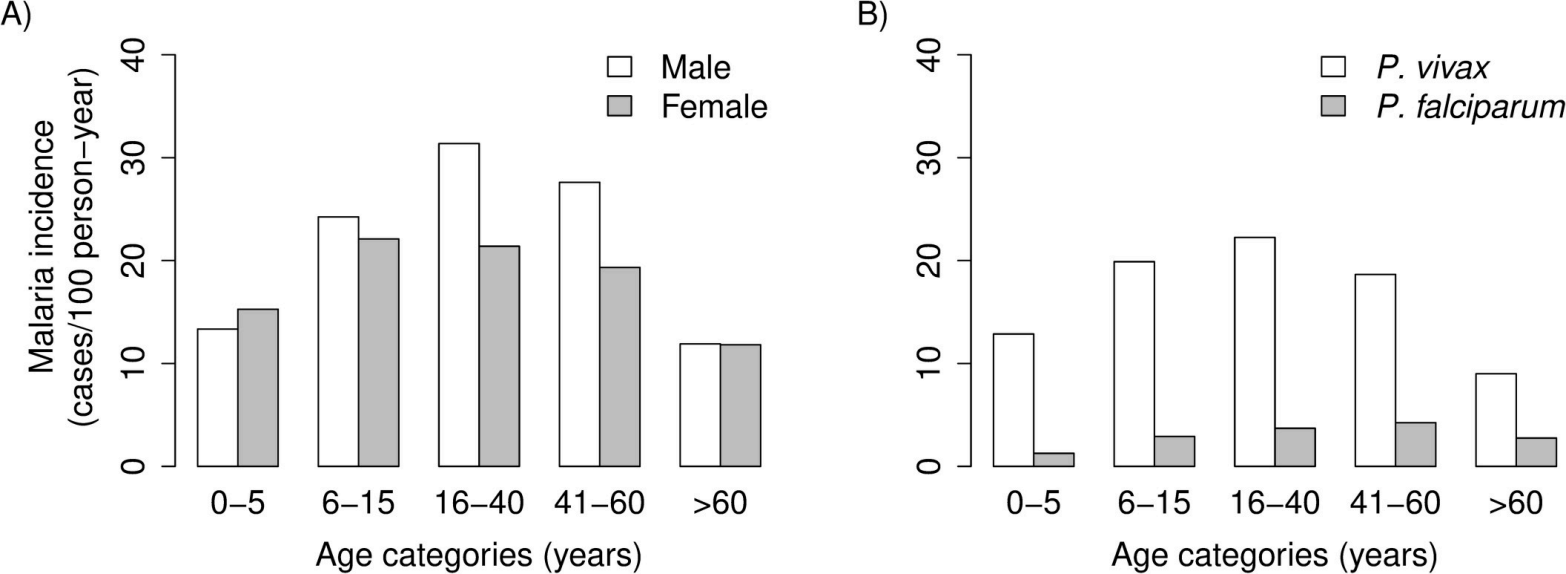
Age-related malaria incidence distribution in urban Mâncio Lima, northwestern Brazil. (A) Age- and gender-related malaria incidence density, regardless of the infecting parasite species. (B) Species-specific age-related malaria incidence density.

The study comprised 8,878 subjects with ages ranging between <1 month and 105 years (mean, 27.0 years) distributed into 2,329 households. They experienced a total of 5,480 laboratory-confirmed malaria episodes over 23,975.3 person-years of follow-up, with an overall malaria incidence density estimated at 22.6 cases per 100 person-years at risk. *Plasmodium vivax* accounted for 84.2% of the episodes (incidence density, 19.0 cases per 100 person-years at risk); 14.4% of the infections were due to *P*. *falciparum*, (incidence density, 3.2 cases per 100 person-years at risk), and 1.4% due to both species. The incidence densities were lowest among under-five children and over-sixty adults (Fig 1A), mostly due to the age-related variation in *P*. *vivax* incidence (Fig 1B). This age-incidence pattern likely reflects the combined effect of differential exposure and acquired immunity across age groups. Male adults aged 16–60 years were more often infected than their female counterparts (Fig 1A), consistent with increased occupational exposure.

### Statistical model fitting

**Fig 2:**
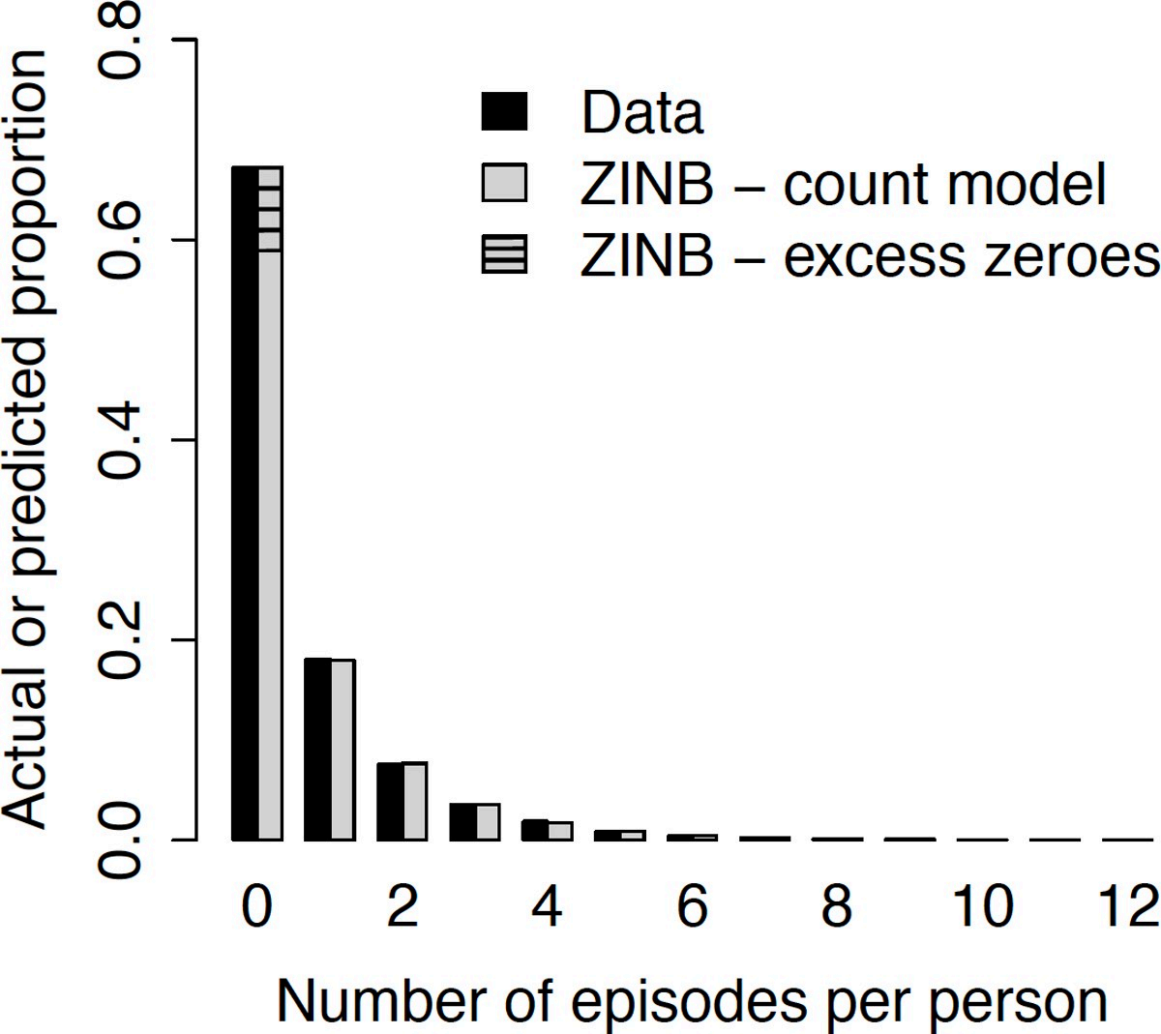
Zero-inflated negative binomial (ZINB) model fit to malaria episode counts per person in urban Mâncio Lima, northwestern Brazil.

The frequency distribution of malaria cases was overdispersed, with a mean of 0.62 (range, 0 to 12; variance, 1.4) episodes per person. The vast majority (67.4%) of study participants remained free of malaria and less than 1% of them had six or more repeated episodes during the follow-up. Empirical frequency distribution data were properly fitted with ZINB distributions (Fig 2).

We analyzed data from 8,431 individuals (447 were excluded due to missing values in key variables) and the RE-ZINB count regression model obtained comprises the explanatory variables listed in S1 Table. RE-ZINB regression analysis estimated that 13.6% (95% CI, 5.1–31.3%) of the study participants (roughly 1,200 residents) were intrinsically free of malaria risk and accounted for the excess zero counts beyond the NB expectations.

**Fig 3:**
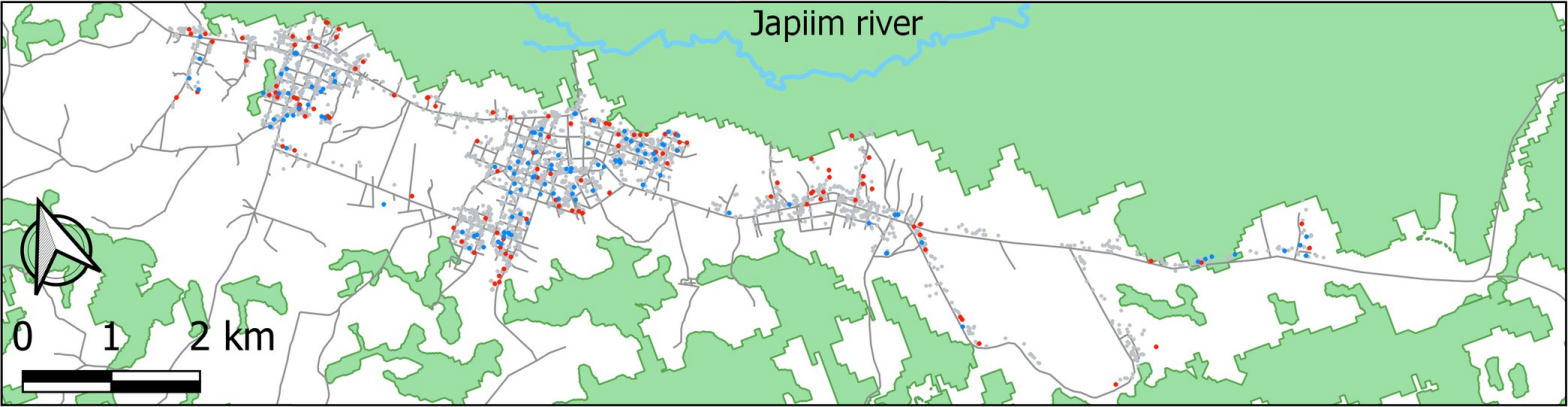
Spatial distribution of households and malaria incidence in urban Mâncio Lima, northwestern Brazil.

We next examined the spatial distribution of “hot houses” and “cold houses”. These were defined as the households within the top 5% (hot houses) and the bottom 5% (cold houses) estimates of random-effects predictors for the count compartment of the RE-ZINB regression model, adjusted for all explanatory variables shown in S1 Table. We show that most hot houses are indeed situated in the periphery of the town (Fig 3) and, therefore, geo-localized houses were classified as centrally or peripherally situated using the computational method described in S3 File. The covariate indicating the zone of residence (whether in the center or in the less-urbanized periphery of the town) was introduced to the regression and the RE-ZINB model fitting was improved (Table 1). These results further indicate that households in the less-urbanized periphery of the town, surrounded by more densely vegetated areas, constitute the priority target for spatial interventions aimed to reduce local malaria transmission.

**Table 1:** Degrees of freedom and Akaike information criterion (AIC) values for the RE-ZINB regression models fitted to empirical data.

Regression model	Zone of residence	Degrees of freedom		AIC
		Fixed effects	Random effects	
Random-effects ZINB	No	17	150	17319.74
	Yes	18	150	17225.61

Study households with lower-than-average (“cold houses”) and higher-than-average malaria incidence (“hot houses”) were identified using the random-effect predictors from the zero-inflated negative binomial (RE-ZINB) model. Red dots show “hot houses” with the top 5% random-effect predictors and blue dots show “cold houses” with the bottom 5% random-effect predictors of RE-ZINB model; all other households are represented as grey dots. Vegetated areas (data retrieved from Brazilian Institute for Space Research (2018) PRODES Project, http://www.inpe.br/cra/projetos_pesquisas/terraclass2014.php.) are shown in green and roads and streets (data retrieved under the Open Database License from the Open Street Map Foundation website at https://www.openstreetmap.org/#map=13/-7.6220/-72.8960&layers=HNas) are shown as thin black lines. Figure created with the QGIS software version 3.4.3, an open source Geographic Information System (GIS) licensed under the GNU General Public License (https://qgis.org/en/site/about/index.html).

### Predictors of malaria incidence density

Table 2 shows independent associations between explanatory variables and malaria incidence density revealed by the best-fitting multivariable ZINB regression model with RE estimators, which include zone of residence as a covariate. We note that the count compartment of the ZINB model allows for identifying predictors of malaria incidence density in the at-risk fraction (86.4%) of the population. Age between 6 and 60 years, male sex, residence in the less-urbanized periphery, and indicators of poor housing quality were key predictors of increased malaria incidence density in the community (Table 2). It is not surprising that LLIN availability in the household, reported bed net use, and recent IRS were all positively associated with malaria incidence density, given that households perceived to be at increased malaria risk are selectively targeted for LLIN distribution and IRS.

**Table 2:** Independent predictors of malaria incidence density in urban Mâncio Lima, Brazil, identified by multivariable random-effects zero-inflated negative binomial (RE-ZINB) regression analysis.

		RE-ZINB model estimates, count compartment			
Variable	No. subjects	IRR	95% CI		P-value
**Individual-level**					
Age (years)					
0–5	1002	Ref.			
6–15	1973	1.29	1.10	1.51	0.0017
16–40	3545	1.51	1.30	1.75	<0.0001
41–60	1262	1.35	1.14	1.60	0.0006
>60	649	0.74	0.59	0.91	0.0053
Gender					
Male	4184	Ref.			
Female	4247	0.79	0.74	0.85	<0.0001
Bed net use the previous night					
No	2469	Ref.			
Yes	5962	1.10	1.01	1.21	0.0328
Follow-up duration					
	8431	3.83	2.71	5.40	<0.0001
**Household-level**					
Zone of residence					
Center	5296	Ref.			
Periphery	3135	1.56	1.44	1.68	<0.0001
Household size					
≤ 5	4524	Ref.			
> 5	3907	1.11	1.03	1.20	0.0084
LLIN available					
No	2870	Ref.			
Yes	3057	1.11	1.02	1.22	0.0196
Unknown	2504	0.95	0.86	1.05	0.2857
Recent IRS					
No	1520	Ref.			
Yes	1497	1.23	1.09	1.39	0.0012
Unknown	5414	1.00	0.90	1.11	0.9724
Complete walls					
No	22	Ref.			
Yes	8409	0.28	0.16	0.49	<0.0001
Type of lavatory					
Outhouse	4657	Ref.			
Indoors	3774	0.86	0.79	0.93	0.0002

Abbreviations: RE-ZINB, random-effects zero-inflated negative binomial; IRR, incidence rate ratio; CI, confidence interval; LLIN, long-lasting insecticidal bed net; IRS, indoor residual spraying.

To further characterize high-risk study participants, we tested whether their increased malaria incidence density was due to larger proportions of subjects experiencing at least one malaria episode or to an increased number of repeated malaria episodes (that may include parasite recrudescences and relapses in addition to new infections) among those who had malaria episodes recorded during the study. We found that both factors contribute to the increased malaria incidence density observed in high-risk population strata. Indeed, 742 (42.5%) of 1,746 male study participants aged 16–40 years, but only 2,020 (30.2%) of the remaining 6,685 study participants, had at least one malaria episode during the 33-month follow-up (*P* < 0.0001, χ^2^ = 94.78, 1 degree of freedom). Moreover, 1,263 (40.3%) of 3,135 study participants living in the periphery of Mâncio Lima, compared to 1,499 (28.3%) of the 5,296 individuals living in the central area of the town, experienced at least one malaria episode during the follow-up (*P* < 0.0001, χ^2^ = 128.36). However, once infected high-risk subjects were also more likely to have repeated malaria episodes during the follow-up. In fact, the frequency distributions of malaria episodes in male study participants aged 16–40 years and those living in the periphery were significantly shifted to the right, compared to their respective counterparts (S2 Fig.).

### Not-at-risk subjects

The not-at-risk fraction of the population described by the structural zero compartment of the RE-ZINB model may be either unexposed, naturally unsusceptible to infection, or may have acquired immunity over time. Because our explanatory variables did not directly measure natural susceptibility or acquired immunity, we focus further analyses on age, sex and zone of residence as proxies of exposure. These variables were added to the logistic (structural zero) component of the RE-ZINB model, which was further adjusted for follow-up duration (person-years at risk). The best-fitting RE-ZINB regression model revealed a negative association of age between 16 and 40 years (but not sex) and residence in the periphery of the town with the odds of being a structural zero. Interestingly age > 60 years (a proxy of cumulative exposure and acquired immunity) remained as a significant predictor of decreased malaria incidence density, but not of being a structural zero (Table 3). This indicates that age-related and spatial differences in malaria exposure, rather than acquired immunity, can account, at least in part, for the presence of not-at-risk subjects in the community. Overall, associations between covariates and malaria incidence density identified by the NB compartment of the RE-ZINB model that also included covariates in the logit compartment (Table 3) were similar to those identified by the RE-ZINB model with an empty (i.e., no covariates added) logit compartment (Table 2).

**Table 3:** Independent predictors of malaria incidence density and odds of being at no risk of malaria in urban Mâncio Lima, Brazil, identified by multivariable random-effects zero-inflated negative binomial (RE-ZINB) regression analysis with explanatory added to the structural zero component of the model.

		Count model				Structural zero			
Variable	No. subjects	IRR	95% CI		P-value	OR	95% CI		P-value
**Individual-level**									
Age (years)									
0–5	1002	Ref.				Ref.			
6–15	1973	1.16	0.93	1.46	0.1892	0.68	0.39	1.20	0.1831
16–40	3545	1.16	0.94	1.44	0.1658	0.31	0.15	0.64	0.0015
41–60	1262	1.15	0.90	1.47	0.2567	0.55	0.28	1.06	0.0745
>60	649	0.63	0.45	0.89	0.0084	0.56	0.19	1.69	0.3046
Gender									
Male	4184	Ref.							
Female	4247	0.78	0.73	0.84	<0.0001				
Bed net use the previous night									
No	2469	Ref.							
Yes	5962	1.11	1.01	1.21	0.0265				
Follow-up duration									
	8431	-	-	-	-	0.13	0.07	0.24	<0.0001
**Household-level**									
Zone of residence									
Center	5296	Ref.				Ref.			
Periphery	3135	1.39	1.25	1.54	<0.0001	0.56	0.38	0.82	0.0031
Household size									
≤ 5	4524	Ref.							
> 5	3907	1.10	1.02	1.19	0.0145				
LLIN available									
No	2870	Ref.							
Yes	3057	1.11	1.01	1.21	0.0276				
Unknown	2504	0.96	0.87	1.06	0.4064				
Recent IRS									
No	1520	Ref.							
Yes	1497	1.22	1.08	1.38	0.0015				
Unknown	5414	0.99	0.90	1.10	0.8864				
Complete walls									
No	22	Ref.							
Yes	8409	0.29	0.16	0.51	<0.0001				
Type of lavatory									
Outhouse	4657	Ref.							
Indoors	3774	0.87	0.80	0.94	0.0003				

Abbreviations: RE-ZINB, random-effects zero-inflated negative binomial; IRR, incidence rate ratio; CI, confidence interval; OR, odds ratio; LLIN, long-lasting insecticidal bed net; IRS, indoor residual spraying.

## Discussion

The long-standing consensus that malaria transmission is spatially heterogeneous provides the basis for targeting control interventions in elimination settings [38,39]. Residual malaria transmission clusters at different spatial scales, from regions to households [40–42], with specific high-risk groups termed “hot-pops” being disproportionally affected [40]. Identifying hot-pops is a top priority of malaria elimination programs.

Here, we examine the drivers of small-area variation in malaria rates in the main urban hotspot in Brazil by fitting multivariable RE-ZINB regression models to community-wide surveillance data. We show that RE-ZINB models can: (i) properly fit overdispersed malaria count data and identify hot pops, (ii) characterize spatial heterogeneity in malaria risk while controlling for potential confounders and identify hot houses, and (iii) characterize the not-at-risk fraction of the population.

Results suggest both imported and locally acquired infections contribute to the malaria burden in the study population. Each entails different malaria control interventions. We hypothesize that increased occupational exposure characterizes the main malaria hot-pop in the community, comprised of adult male residents often engaged in subsistence farming in nearby settlements [43]. These subjects may serve as a source of new parasite strains continuously introduced in the town, being the main targets of interventions to reduce malaria importation. Control measures may include deploying periodic malaria screening and treatment, as well as LLINs, to the most mobile subjects in the community. Conversely, the RE-ZINB model estimates that 14% of the study participants comprises the not-at-risk fraction of the population. This relatively large fraction of the urban population is mostly comprised of children and older adults living in the central urban hub who will remain uninfected regardless of any malaria control measure.

Local transmission also appears to contribute to malaria risk, especially in the less-urbanized periphery. We confirm that better housing is associated with reduced malaria incidence [44,45] even in an endemic setting dominated by vectors that feed and rest predominantly outdoors [46]. Interestingly, hot houses identified by the analysis of random-effects predictors of the RE-ZINB regression model tend to be peripherally located, but they do not form clear, easily detectable clusters. Importantly, the fraction of study participants residing along the town boundaries (37% of the total) appear to be at increased risk after controlling for potential confounders, indicating that the association between place of residence and malaria risk is mostly spatial, and is not severely confounded by age, sex, and housing quality differences among households. These findings are consistent with focal malaria transmission across the urban-rural transition in the periphery of the town [43]. Control measures to reduce local malaria transmission include, among others, IRS and LLIN distribution targeted at hot houses. Moreover, large-scale screening of windows and other house openings may represent a valuable measure to render high-risk hot houses mosquito-proof, as suggested by recent data from urban Africa [47].

The present study has some limitations. First, surveillance data were retrieved retrospectively from a case notification database and no blood samples were available for further confirmatory (e.g., molecular) diagnostic tests. We assume that nearly all malaria episodes diagnosed by microscopy and treated in study participants were retrieved [32], but routine surveillance overlooks transient sub-microscopic parasitemias that do not develop into detectable infections but remain infectious to mosquitoes [48]. Therefore, risk factors described for microscopy-positive malaria in the community are not necessarily the same for sub-microscopic, often asymptomatic infections. Next, surveillance data comprises cases diagnosed by both passive and active case detection, but our data set does not allow for distinguishing between case-finding strategies. Moreover, analyses of passively detected cases are prone to biases due to variation in access to health facilities and health-seeking behavior, even in relatively compact urban areas where health facilities are readily accessible and provide care at no cost. Finally, the infrequency of *P*. *falciparum* malaria precludes further between-species comparisons of risk factors in the study population.

## Conclusion

We conclude that both local transmission and imported cases from rural and/or forest areas are responsible for the maintenance of malaria in the urban setting of Mâncio Lima. Large sets of routinely collected surveillance data linked to additional demographic and socioeconomic information can be explored for evidence-based planning and deployment of malaria control interventions.

## Supporting information

S1 Fig Mâncio Lima. Map of South America showing the location of the field site, the municipality of Mâncio Lima in the state of Acre, northwestern Brazil, next to the border with Peru. Figure created using data extracted from the GADM database (www.gadm.org), version 2.8, under the Creative Commons Attribution License (CCAL) CC BY 4.0 (http://creativecommons.org/licenses/by/4.0/).
(TIF)

S2 Fig Number of malaria episodes per person in different population strata in urban Mâncio Lima, northwestern Brazil. Only study participants who had at least one malaria episode diagnosed during the follow-up are included in this analysis. The upper panel shows the frequency distributions of malaria episodes in males aged 16–40 years (A; n = 742 study participants) and in all other population strata in Mâncio Lima (B; n = 2,020). The frequency distributions are significantly different (Kolmogorov-Smirnov test, *P* = 0.0219). The lower panel shows the frequency distributions of malaria episodes in study participants living in the periphery (C; n = 1,263) and in the center (D; n = 1,499) of the town of Mâncio Lima. The frequency distributions are also significantly different (Kolmogorov-Smirnov test, *P* < 0.0001).
(TIF)

S1 File Improving the fitting of RE-ZINB models by shrinking the random-effects predictors toward their overall mean. (DOCX)

S2 File Testing for associations between covariates included in the RE-ZINB models. (DOCX)

S3 File Computational procedure to delineate the study site boundaries and classify households as centrally or peripherally located. (DOCX)

S1 Table Independent predictors of malaria incidence in urban Mâncio Lima, Brazil, identified by multivariable RE-ZINB regression analysis without the spatial covariate (zone of residence). (DOCX)

## Abbreviations

AIC: Akaike information criterion; CI, confidence interval
GAIC: generalized Akaike information criterion
GAMLSS: generalized additive models for location, scale and shape
IRR: incidence rate ratio
IRS: indoor residual spraying
LLIN: long-lasting insecticidal bed net
OR: odds ratio
Q-Q plot: quantile-quantile plot
RE: random effects
ZINB: zero-inflated negative binomial
RE-ZINB: random-effects zero-inflated negative binomial
