# Supplementary figures and images for: Statistical modeling of surveillance data to identify correlates of urban malaria risk: A population-based study in the Amazon Basin
Source: PLoS One. 2019 Aug 9;14(8):e0220980. doi: 10.1371/journal.pone.0220980 (PMC6688813; doi:10.1371/journal.pone.0220980)

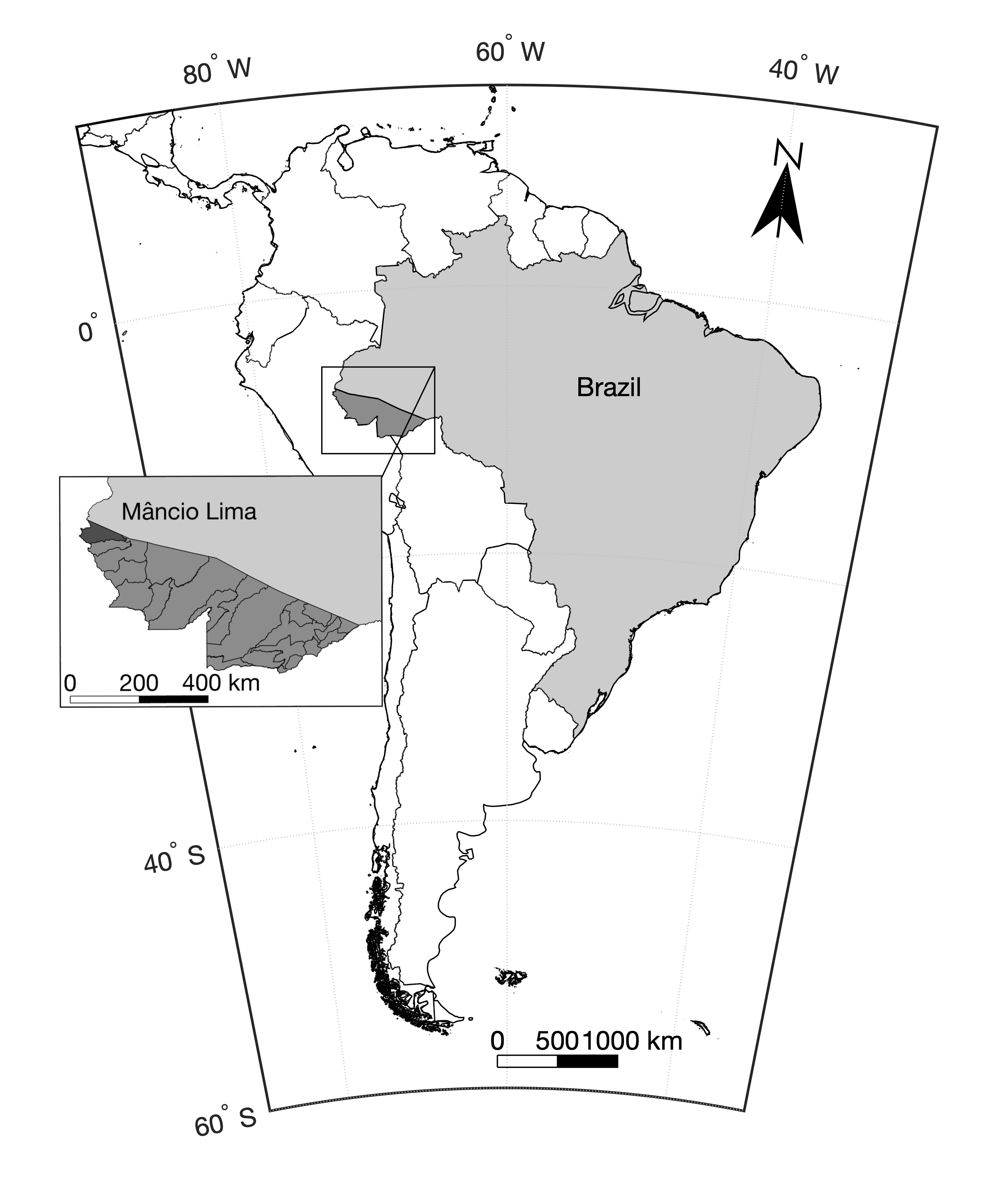

Supplement: S1 Fig — Map of South America showing the location of the field site, the municipality of Mâncio Lima in the state of Acre, northwestern Brazil, next to the border with Peru. Figure created using data extracted from the GADM database (www.gadm.org), version 2.8, under the Creative Commons Attribution License (CCAL) CC BY 4.0 (http://creativecommons.org/licenses/by/4.0/). (TIF) [file pone.0220980.s001.tif]

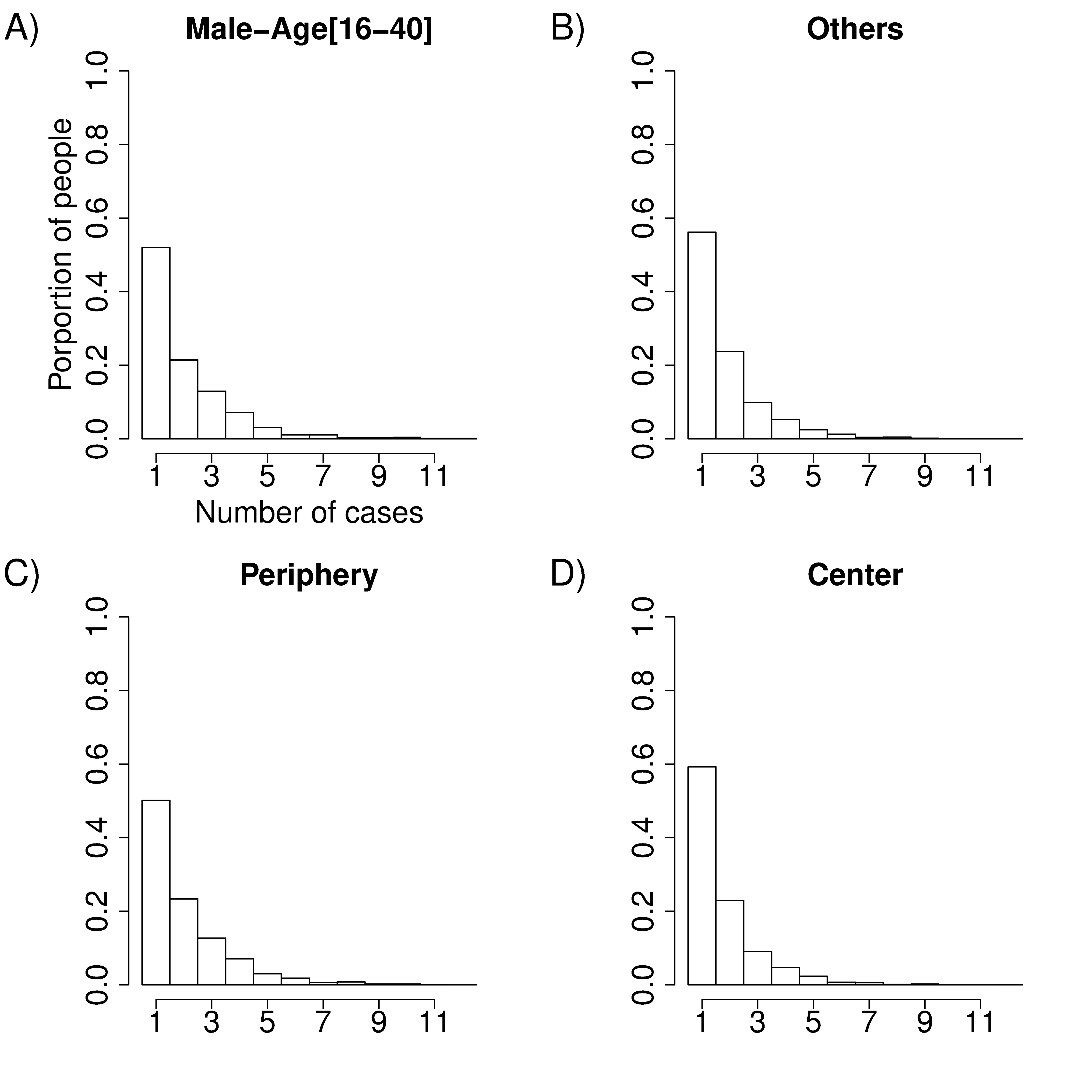

Supplement: S2 Fig — Only study participants who had at least one malaria episode diagnosed during the follow-up are included in this analysis. The upper panel shows the frequency distributions of malaria episodes in males aged 16–40 years (A; n = 742 study participants) and in all other population strata in Mâncio Lima (B; n = 2,020). The frequency distributions are significantly different (Kolmogorov-Smirnov test, P = 0.0219). The lower panel shows the frequency distributions of malaria episodes in study participants living in the periphery (C; n = 1,263) and in the center (D; n = 1,499) of the town of Mâncio Lima. The frequency distributions are also significantly different (Kolmogorov-Smirnov test, P < 0.0001). (TIF) [file pone.0220980.s002.tif]
