# Supplementary material for: Statistical modeling of surveillance data to identify correlates of urban malaria risk: A population-based study in the Amazon Basin
Source: PLoS One. 2019 Aug 9;14(8):e0220980. doi: 10.1371/journal.pone.0220980 (PMC6688813; doi:10.1371/journal.pone.0220980)
Supplement: S1 File — (DOCX) [file pone.0220980.s003.docx]

**S1 file: Improving the fitting of RE-ZINB model by shrinking the random-effects predictors toward their overall mean**

The worm plots corresponding to the RE-ZINB regression initially showed many data points lying outside the 95% confidence intervals (CI) due to too large fitted variance, as indicated by the negative slope of the worm-like string in the upper panel of Fig S1. To reduce the fitted variance of the model, we shrunk the random-effects predictors toward their overall mean by limiting their degrees of freedom originally estimated by the model. For the multivariable RE-ZINB model, we obtained the most satisfactory, nearly horizontal worm-like strings by decreasing the number of degrees of freedom of the random-effects predictors to 150 (Fig S1, bottom panel).

**Fig S1.** Diagnostic worm plot and randomized normal quantile-quantile (Q-Q) plots for residual analysis and random-effect distribution for the RE-ZINB model with different values of random-effect degrees of freedom. Black dots in worm plots represent the average of 20 model realizations; individual data points for each realization are shown as open grey circles. The dashed black lines delimit the 95% confidence interval.
