# Supplementary material for: Statistical modeling of surveillance data to identify correlates of urban malaria risk: A population-based study in the Amazon Basin
Source: PLoS One. 2019 Aug 9;14(8):e0220980. doi: 10.1371/journal.pone.0220980 (PMC6688813; doi:10.1371/journal.pone.0220980)
Supplement: S2 File — (DOCX) [file pone.0220980.s004.docx]

**S2 file. Testing for associations between covariates included in the RE-ZINB models**

We explored possible associations between covariates included in the RE-ZINB models by using the Goodman and Kruskal tau measure (asymmetric association measure between categorical variables) as implemented in the R package *GoodmanKruskal*. We found no significant pairwise association between the covariates.

The following covariates were tested:

**Individuals**

Age cov.01

Gender cov.02

Bednet use the previous night cov.03

Sleeping time cov.04

Waking-up time cov.05

**Household**

Household size cov.06

Wealth index cov.07

LLIN available cov.08

IRS within the past three years cov.09

Incomplete walls cov.10

Ceiling cov.11

Door screens cov.12

Windows screens cov.13

Type of lavatory cov.14

Zone of residence cov.15


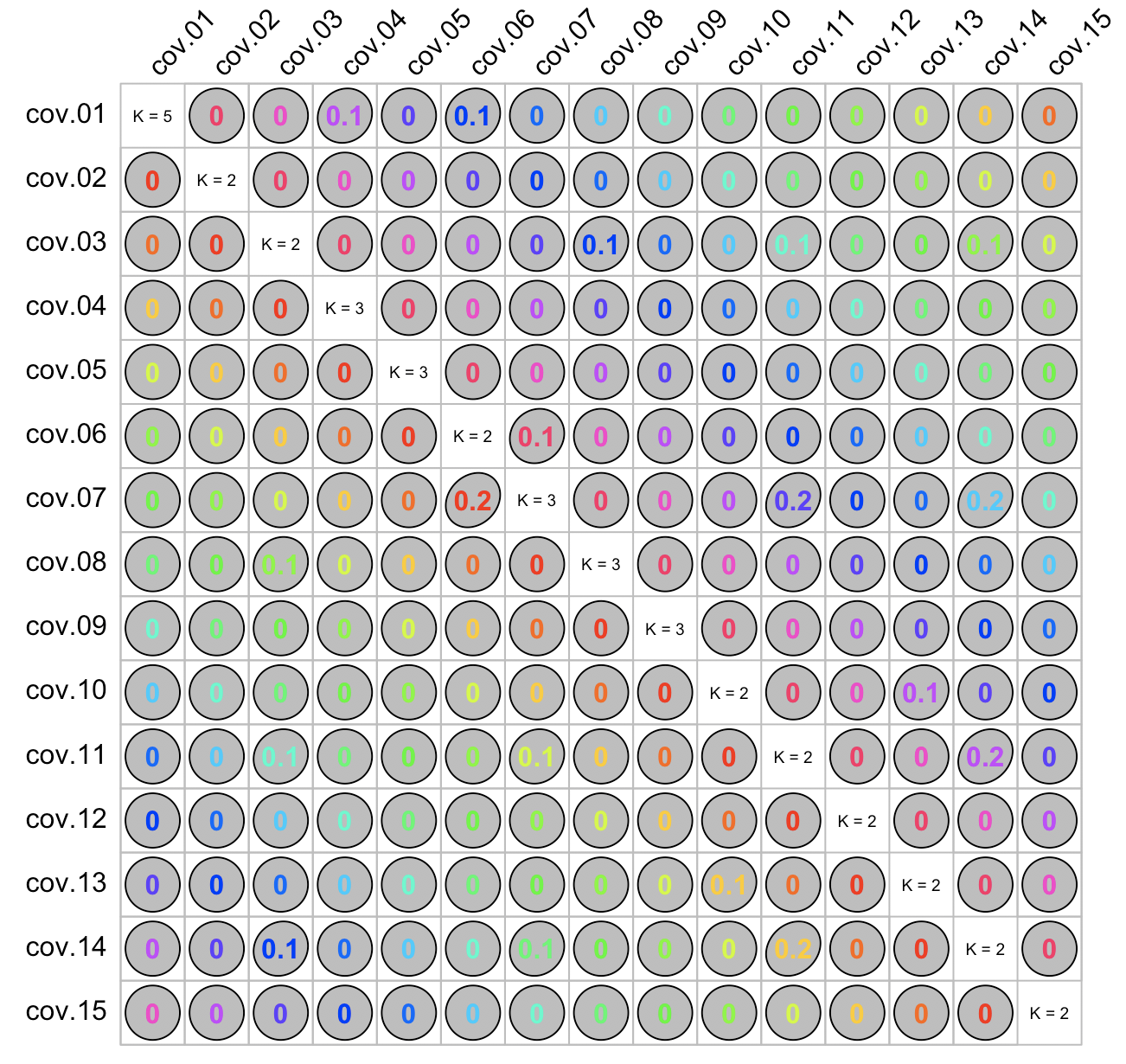


**Fig S1.** Goodman and Kruskal tau association measure between categorical model covariates. The diagonal entries give the numbers of unique levels for each variable, while the off-diagonal elements give both numeric and graphical representations of the Goodman-Kruskal τ values. Specifically, the numerical values appearing in each row represent the association measure τ(x,y) from the variable x indicated in the row name to the variable y indicated in the column name. Note that this matrix is generally not symmetric, in contrast to standard correlation matrices. All τ values obtained are ≤ 0.2, suggesting that covariates exhibit a slight ability to explain variation in the other covariates.
