# Supplementary material for: Statistical modeling of surveillance data to identify correlates of urban malaria risk: A population-based study in the Amazon Basin
Source: PLoS One. 2019 Aug 9;14(8):e0220980. doi: 10.1371/journal.pone.0220980 (PMC6688813; doi:10.1371/journal.pone.0220980)
Supplement: S3 File — (DOCX) [file pone.0220980.s005.docx]

**S3 file. Computational procedure to delineate the study site boundaries and classify households as centrally or peripherally located**

A procedure for the graphical definition of the town boundaries was implemented in Matlab software to classify households according to their localization, whether in the less urbanized periphery or in the center of the town. First, we generated the so-called alpha shape [1] with a two-dimension set of points given by the GPS coordinates of each household (Fig S1A and S1B) The alpha shape is a generalization of the convex hull of a finite set of points. A convex hull is the smallest convex region enclosing all points in the set (for more details, see [2]). An alpha shape is also a subgraph of the Delaunay triangulation [3] which is controlled by a parameter (alpha radius) that ranges from 0 to infinite and determines the level of boundary detail. The smaller the alpha radius, the higher the number of points at the boundary.


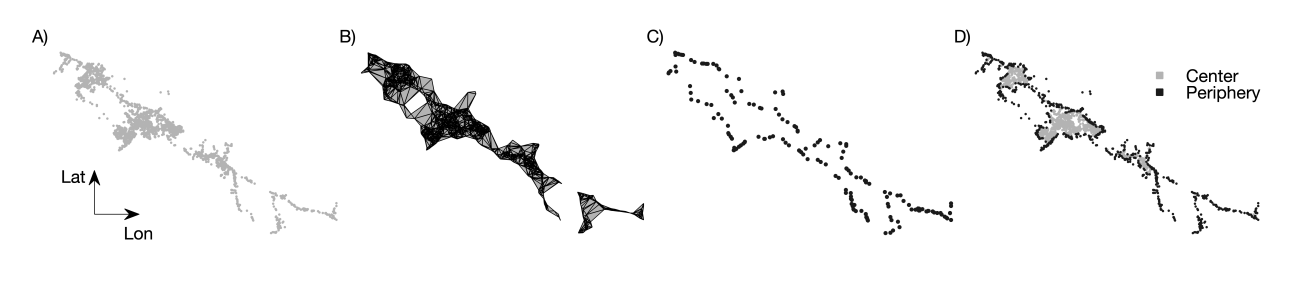


**Fig S1.** Computational procedure to delineate the study site boundaries and classify households as centrally and peripherally located. (A) Two-dimension set of points given by the GPS coordinates of each household from the city of Mâncio Lima. (B) Alpha shape from the two-dimension set of points given by the GPS coordinates of each household. (C) Boundary points defined by the alpha shape from the two-dimension set of points given by the GPS coordinates of each household. (D) Classification of zone of residence for the city of Mâncio Lima as center (light gray dots) and periphery (black dots) by using alpha shapes with alpha radiuses equal to 0.004, 0.003, 0.002 and 0.00135.

We generated an alpha shape with a defined alpha radius and selected households that connect facets at the boundary of the alpha shape (Fig S1C). With the remaining households (i.e., those not classified as peripherally located in the first step), we generated a new two-dimension set of points given by their GPS coordinates. Due to the shape of the city, the above strategy was applied four times, with the following values for the alpha radius: 0.004, 0.003, 0.002 and 0.00135. Figure S1D shows the distinction between periphery and center of the town defined by the procedure, which was used to create the covariate “zone of residence” used in regression analysis.

**References**

[1] Edelsbrunner H, Kirkpatrick D, Seidel R. On the shape of a set of points in the plane. IEEE Trans Inform Theory 1983;29:551-9.

[2] de Berg M, van Kreveld M, Overmars M, Schwarzkopf O. Computational Geometry: Algorithms and Applications. 2nd. edition. Berlin: Springer; 2000.

[3] Delaunay B. Sur la sphère vide. A la mémoire de Georges Voronoï. Izv Akad Nauk SSSR, Otdelenie Matematicheskih i Estestvennyh Nauk 1934;7:793–800.
