# Supplementary material for: Statistical modeling of surveillance data to identify correlates of urban malaria risk: A population-based study in the Amazon Basin
Source: PLoS One. 2019 Aug 9;14(8):e0220980. doi: 10.1371/journal.pone.0220980 (PMC6688813; doi:10.1371/journal.pone.0220980)
Supplement: S1 Table — (DOCX) [file pone.0220980.s006.docx]

**S1 Table. Independent predictors of malaria incidence in urban Mâncio Lima, Brazil, identified by multivariable random-effects zero-inflated negative binomial (RE-ZINB) without the spatial covariate (zone of residence).**

|  |  | RE-ZINB model estimates, count compartment | | | |
| --- | --- | --- | --- | --- | --- |
| **Explanatory variable** | **No. subjects** | IRR | 95% CI | | P-value |
| **Individual-level** |  |  |  |  |  |
| Age (years) |  |  |  |  |  |
| 0-5 | 1002 | Ref. |  |  |  |
| 6-15 | 1973 | 1.29 | 1.10 | 1.51 | 0.0016 |
| 16-40 | 3545 | 1.51 | 1.30 | 1.75 | <0.0001 |
| 41-60 | 1262 | 1.34 | 1.13 | 1.58 | 0.0009 |
| >60 | 649 | 0.73 | 0.59 | 0.91 | 0.0050 |
| Gender |  |  |  |  |  |
| Male | 4184 | Ref. |  |  |  |
| Female | 4247 | 0.79 | 0.74 | 0.86 | <0.0001 |
| Bed net use the previous night |  |  |  |  |  |
| No | 2469 | Ref. |  |  |  |
| Yes | 5962 | 1.15 | 1.05 | 1.26 | 0.0020 |
| Follow-up duration |  |  |  |  |  |
|  |  | 3.89 | 2.75 | 5.50 | <0.0001 |
| **Household-level** |  |  |  |  |  |
| Household size |  |  |  |  |  |
| ≤ 5 | 4524 | Ref. |  |  |  |
| > 5 | 3907 | 1.12 | 1.04 | 1.21 | 0.0047 |
| LLIN available |  |  |  |  |  |
| No | 2870 | Ref. |  |  |  |
| Yes | 3057 | 1.18 | 1.08 | 1.29 | 0.0004 |
| Unknown | 2504 | 0.97 | 0.88 | 1.08 | 0.5993 |
| Recent IRS |  |  |  |  |  |
| No | 1520 | Ref. |  |  |  |
| Yes | 1497 | 1.32 | 1.16 | 1.49 | <0.0001 |
| Unknown | 5414 | 1.02 | 0.92 | 1.13 | 0.6824 |
| Ceiling |  |  |  |  |  |
| No | 6666 |  |  |  |  |
| Yes | 1765 |  |  |  |  |
| Complete walls |  |  |  |  |  |
| No | 22 | Ref. |  |  |  |
| Yes | 8409 | 0.34 | 0.19 | 0.60 | 0.0002 |
| Type of lavatory |  |  |  |  |  |
| Outhouse | 4657 | Ref. |  |  |  |
| Indoors | 3774 | 0.83 | 0.77 | 0.90 | <0.0001 |

Abbreviations: RE-ZINB, random-effects zero-inflated negative binomial; IRR, incidence rate ratio; CI, confidence interval; LLIN, long-lasting insecticidal bed net; IRS, indoor residual spraying.
